# Peroxisome Proliferator-Activated Receptor-γ Antagonizes LOX-1-Mediated Endothelial Injury by Transcriptional Activation of miR-590-5p

**DOI:** 10.1155/2019/2715176

**Published:** 2019-07-01

**Authors:** Lei Xu, Gang Zhao, Hong Zhu, Shijun Wang, Aijun Sun, Yunzeng Zou, Junbo Ge

**Affiliations:** ^1^Shanghai Institute of Cardiovascular Diseases, Zhongshan Hospital, Fudan University, 180 Fenglin Road, Shanghai 200032, China; ^2^Laboratory of Oral Microbiology, Shanghai Research Institute of Stomatology, Shanghai Key Laboratory of Stomatology, Ninth People's Hospital, School of Stomatology, Shanghai Jiao Tong University School of Medicine, Shanghai 200011, ChinaChina

## Abstract

Lectin-like oxidized low-density lipoprotein receptor-1 (LOX-1) is one of the major receptors expressed on the endothelium of arterial wall with a key role in endothelial dysfunction and the development of atherosclerosis. Recent evidence suggested that LOX-1 is upregulated under the condition of insulin resistance and could be suppressed by the antidiabetic drugs. We previously also confirmed that Thiazolidinedione (TZD) has the inhibitory effect on LOX-1 in ox-LDL-induced endothelial cells. However, the underlying mechanism is unclear. Here we showed that Rosiglitazone treatment significantly attenuated the expressions of LOX-1, ICAM-1, VCAM-1, p47^phox^, and the atherosclerotic lesions in ApoE^−/−^ mice with high-fat diet. In vitro, we revealed that Rosiglitazone inhibited LOX-1 by regulating miR-590-5p. Ox-LDL-mediated ICAM-1, VCAM-1, and p47^phox^ were significantly reduced by Rosiglitazone, but all reversed after pretreating the cells with antagomiR-590-5p. Induction with Rosiglitazone activated PPAR-*γ* and promoted its nuclear translocation in cultured human umbilical vein endothelial cells (HUVECs). The nuclear PPAR-*γ* upregulated the miR-590-5p level through binding to its transcriptional promoter region. Retaining PPAR-*γ* in cytoplasm by transfecting with PPAR-γ^⊿NLS^ plasmid in HUVECs failed to activate miR-590-5p. Mutation of the promoter region of PPAR-*γ* also reduced the miR-590-5p promoter luciferase activity. Collectively, these data indicated that PPAR-*γ* may have the therapeutic potential in atherosclerosis via the transcriptional regulation of miR-590-5p in endothelial cells.

## 1. Introduction

Endothelial oxidative injury is considered as the leading cause of coronary atherosclerosis, and oxidized low-density lipoprotein cholesterol (ox-LDL) is critically involved in endothelial oxidative injury and dysfunction [1]. Lectin-like oxidized low-density lipoprotein receptor-1 (LOX-1) is the major scavenger receptor for Ox-LDL, which promotes the uptake of ox-LDL by endothelial cells (ECs) in the arterial wall, and accelerates the process of inflammation and oxidative stress [1, 2]. The basal line of LOX-1 in ECs is very low, but it can be rapidly induced by prooxidative stress, such as ox-LDL, angiotensin II (AngII), advanced glycation end productions (AGEs), and proinflammatory cytokines [2–4]. The activation of LOX-1 promotes EC uptake of large amounts of ox-LDL, leads to apoptosis of vascular endothelial and smooth muscle cells, increases the production of matrix metalloproteinases and intercellular adhesion molecules, which promotes migration and infiltration of inflammatory cells, and accelerates the atherosclerotic progression and plaque vulnerability [2, 5].

Peroxisome proliferator activated receptors (PPARs) belong to the superfamily of ligand-activated transcription factors, including PPAR-*α*, PPAR-*β*/*δ*, and PPAR-*γ* three subtypes [6, 7]. Different from the others, PPAR-*γ* is mainly expressed in adipocytes and vascular endothelial cells [8]. The wide spectrum effects of PPAR-*γ* activation may be beneficial for lipid metabolism, promoting free fatty acid *β*-oxidation, reducing the accumulation of plasma triglycerides, and preventing the development of atherosclerosis [7, 9]. Abnormal fat distribution and partial lipodystrophy are observed in mutant PPAR-*γ* gene patients [10]. In our previous work, we showed that ox-LDL-mediated endothelial LOX-1 upregulation was suppressed by Ciglitazone, a special PPAR-*γ* agonist. However, the underlying mechanism is unclear.

Previous studies indicated that ox-LDL-induced LOX-1 activation could be inhibited by miR-590-5p [11, 12], and miR-590-5p agomir effectively prevented the development of atherosclerosis in ApoE^−/−^ mice through attenuating lipid accumulation and proinflammatory cytokine secretion [13, 14]. Induction with miR-590-5p effectively decreased cellular reactive oxygen species (ROS), blocked the p38MAPK signaling pathway, and inhibited the nuclear translocation of Nf-*κ*B by targeting LOX-1 [9, 12]. Emerging lines of evidence suggested that PPAR-*γ*, as an important nuclear transcriptional factor, promoted the transcription of multiple microRNAs including miR-125a, miR-424, and miR-503, which were critically involved in regulating endothelial-dependent inflammation and angiogenesis [15, 16]. Therefore, in this study we hypothesized that PPAR-*γ* activation might inhibit ox-LDL-induced LOX-1 by targeting miR-590-5p, which improved and even reversed the pathological course of atherosclerosis.

## 2. Methods

### 2.1. Animal Model and Sample Collection

Eight-week old male C57BL/6  ApoE gene knockout (ApoE^−/−^) mice were purchased from Beijing Vital River Laboratory Animal Technology Co., Ltd. All the mice were housed in SPF conditions and randomly divided into three groups, the control group (n=10), the group of ApoE^−/−^ mice (n=10) which only received high-fat diet, and another group (n=10) which received high-fat diet with Rosiglitazone (2mg/kg/day, BRL49653, MCE) by oral gavage daily, respectively, for 10 weeks as previously described [17]. Body weights of all the mice were monitored and recorded for every week. After 10 weeks, all the mice were sacrificed by inhaled anesthesia with 3% isoflurane. The blood sample of each mouse collected by extracting the eyeball blood and the plasma levels of TC and HDL-C were determined by enzymatic method using commercial kits as previously described [18]. The aortas and related tissues were isolated from mice and were quickly fixed for immunohistochemistry slice or frozen in liquid nitrogen for protein analysis.

### 2.2. Quantification of Atherosclerotic Lesion Areas

After the mice were sacrificed, the chest was opened and the proximal aorta attached to the heart was isolated and fixed in 4% paraformaldehyde for 24 h. Cross-section (~7 *μ*m) of the aorta root was collected and stained with oil-red O and brilliant green. The volume of atherosclerotic lesions was calculated from five different sections. Quantitative analysis of stained lipid area was performed by a blinded observer using Leica QWin V3 software.

### 2.3. In Vitro HUVECs Culture and Cell Immunostaining

Primary HUVECs were purchased from AllCells (Shanghai, China) and cultured in low-glucose DMEM with 10% FBS (Gibco). After being in vitro cultured and passaged for 8~10 times, the cells were used for experiments. After treating the cells with or without GW9662 (5 *μ*mol/L) for 1 hour followed by the induction with Rosiglitazone (50 *μ*mol/L) for 24 hours, HUVECs were fixed by 4% paraformaldehyde, permeabilized in 0.1% Triton X-100, and blocked with 3% BSA for 30 min, and then they were stained by anti-PPAR-*γ* (1:200, ab45036, Abcam) for 1 hour at 4°C overnight. After washing for 3 times, the cells were stained by Alexa Fluor® 488-conjugated goat anti-rabbit IgG (1:1000).

### 2.4. Reconstruction and Transfection of the Plasmids

The mutant PPAR-*γ* without the NLS peptide (PPAR-*γ*^*⊿*NLS^) was performed by plasmid reconstruction. The NLS peptide with a core residue “*LSVMDDHSH*” located at the junction between DNA binding domain (DBD) and hinge domain of PPAR-*γ* gene was conserved in different species [19, 20]. This region was deleted in the wild-type (WT) PPAR-*γ* plasmid (Obio Tech, Shanghai) using restriction endonuclease and PCR fragment amplification method. The linear plasmid was recombined with T4 DNA ligase. Both WT PPAR-*γ* and PPAR-*γ*^*⊿*NLS^ were transfected into HUVECs using Lipofectamine LTX with Plus reagent (Life Technologies) according to the manufacturer's instructions.

### 2.5. Western Blot Analysis

HUVECs grown on six-well plate were lysed in RIPA buffer containing the protease inhibitor, 1mM PMSF (ST506, Beyotime Biotech.) on ice for 20 min. Total proteins were extracted from the lysates by centrifugation (11000 rpm, 4°C, 10 min). Then the proteins (20 *μ*g) were loaded onto SDS-PAGE gels (12%) and separated by electrophoresis, followed by transfer onto the PVDF membrane (Bio-Rad). The immunoblots on the membrane were blocked with 5% BSA and then incubated with the primary antibodies (anti-LOX-1, rabbit polyclonal antibody at 1:1000 dilution, ab203246, Abcam; anti-ICAM-1, mouse monoclonal antibody at 1:1000 dilution, ab20, Abcam; anti-VCAM-1, rabbit monoclonal antibody at 1:1000 dilution, ab 174279, Abcam; and anti-p47^phox^, rabbit polyclonal antibody at 1:1000 dilution, #4312, Cell Signaling Technology). After washing with 1×PBST for 3 times, the blots were incubated again with the horseradish peroxidase (HRP) conjugated secondary antibody IgG (1:5000, Kangchen Biotechnology, Shanghai). GAPDH was used as the internal control. The immunoblot complexes were detected by high resolution chemiluminescence Western blot detection reagents (Thermo scientific, 34080) and the images were captured by ChemiDocTM Touch Imaging System (BIO-RAD, CA, USA).

### 2.6. Chromatin Immunoprecipitations (ChIPs)

After transfecting the WT-PPAR-*γ* or PPAR-*γ*^*⊿*NLS^ plasmid into HUVECs, the cells were stimulated by Rosiglitazone (50 *μ*mol/L) for 24 h. Cells were then fixed with 1% formaldehyde for 10 min and stopped by adding glycine to a final concentration of 200 mmol/L. After washing with ice-cold 1×PBS for 3 times, cells were homogenized on ice by a sonicator within cell lysis buffer containing 0.2% NP-40 and protease inhibitors. Then, chromatin DNA were extracted and sheared into ~500 bp fragments. After centrifugation for 5 min, the supernatant was diluted with 10-fold dilution buffer and reserved as negative control. The remaining lysates were precleared with Dynabeads™ Protein A (50 *μ*L) and agitated at 4°C for 30 min. The anti-PPAR-*γ* antibody (1:200, ab45036, Abcam) or a control IgG (1:200) was added into the precleaned supernatant, and then the supernatant was incubated at 4°C overnight with constant rotation. ChIP beads were washed six times at room temperature and incubated with proteinase K for 30 min and reverse cross-linked by heating at 65°C for 4 hours. The immunoprecipitated DNA was purified by phenol/chloroform extraction and recovered for PCR reaction, and the temperature cycling was denaturation at 95°C for 30s, annealing at 56°C for 30s, and extension at 72°C for 30s, and it was repeated for 34 cycles. PCR primers were as follows: the upstream primer: 5′-CCTCTCCTTCCCCTTCTCCT-3′; the downstream primer: 5′-TTAAAGGCTGAACACGGTGG-3′.

### 2.7. Luciferase Report Assay

Promoter luciferase reporter assay was produced by a pMIR-REPORT™ system as described previously [21]. The miR-590-5p promoter regions containing the PPAR-*γ* core binding motif “TAGGTCA” or the mutated type (mut) motif “TAAATAA” were, respectively, amplified and cloned into the pMIR-REPORT Luciferase plasmid, which located at the upstream of the luciferase reporter gene and was driven by the CMV enhancer. Each type of reporter plasmids and pCMV-Renilla control were, respectively, transfected into HEK-293T cells 48 h before stimulating with Rosiglitazone. Cell lysates were collected and the luciferase activities were assayed by Dual-Luciferase Reporter Assay System (Promega, Madison, WI) according to the manufacturer's instructions.

### 2.8. Statistical Analysis

All data are presented as mean ± SEM. Analyses were performed with statistical software SPSS 16.0. The differences among groups were analyzed by One-Way ANOVA of variance followed by LSD analysis. Values were considered statistically significant when P < 0.05.

## 3. Results

### 3.1. Rosiglitazone Suppressed Endothelial LOX-1 in Atherosclerotic ApoE^−/−^ Mice

**Figure 1:**
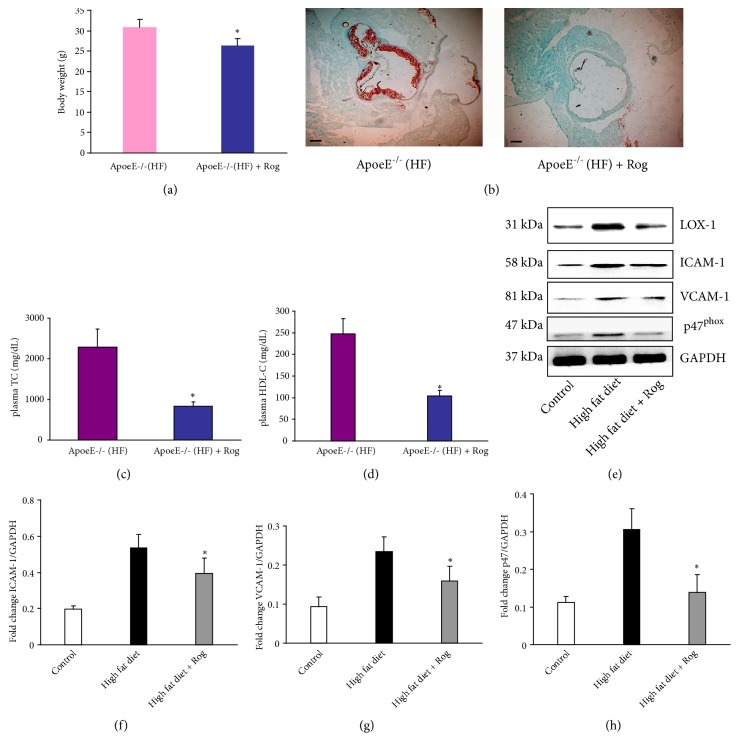
*Preferential induction of Rosiglitazone prevented atherosclerosis in high-fat diet-induced ApoE* ^*-/-*^ *mice*. Male ApoE^−/−^ mice were subjected to high-fat diet with or without Rosiglitazone (2mg/kg/day) for continuous 10 weeks. (a) The body weight of mice in each group (n=10) was recorded at the end of 10 weeks. (b) Representative images of the cross-sectional aortic sinus stained with Oil Red O (scale bar, 100 *μ*m). (c) The plasma levels of total cholesterol (TC) and (d) high density lipoprotein cholesterol (HDL-C) were determined and compared in both groups (*∗* P<0.05). (e) Representative images of protein blots of LOX-1, ICAM-1, VCAM-1, and p47^phox^ in aortic endothelium of the ApoE^−/−^ mice by Western blot. (f–h) Quantitative analysis of the protein levels of LOX-1, ICAM-1, VCAM-1, and p47^phox^ (n=4, *∗* P<0.05 vs. High fat diet group).

To investigate the role of PPAR-*γ* activation in regulating endothelial function in an atherosclerosis model, the ApoE^−/−^ mice were subjected to high-fat diet for 10 weeks with or without Rosiglitazone. The results showed that Rosiglitazone treatment reduced the body weight gain of ApoE^−/−^ mice by 15.2% (Figure 1(a)). Compared with high-fat-induced ApoE^−/−^ mice, plasma levels of TC (721.8±130.4 vs. 2254.3±276.0 mg/dL, P <0.05) and HDL-C (101.3±11.5 vs. 246.5±26.1mg/dL, P <0.05) were both significantly reduced in Rosiglitazone-pretreated mice (Figures 1(c) and 1(d)). Consistent with the abnormal elevation of plasma TC and HDL-C, severe atherosclerotic plaques were observed in the aortic root of coronary artery of ApoE^−/−^ mice, but the atherosclerotic lesions were not obviously augmented in Rosiglitazone pretreated ApoE^−/−^ mice (Figure 1(b)). Of note, the increased protein level of LOX-1 in the coronary endothelium of ApoE^−/−^ mice after high-fat diet was significantly decreased by Rosiglitazone. Accompanied by the upregulation of LOX-1, high-fat diet also increased the expressions of ICAM-1, VCAM-1, and p47^phox^ in ApoE^−/−^ mice, which were all reversed by Rosiglitazone treatment (Figures 1(e)–1(h)), suggesting that PPAR-*γ* activation might effectively suppress LOX-1 expression and prevent endothelial inflammation and oxidative injury during the development of atherosclerosis.

### 3.2. miR-590-5p Was Downregulated in ox-LDL-Induced HUVECs

**Figure 2:**
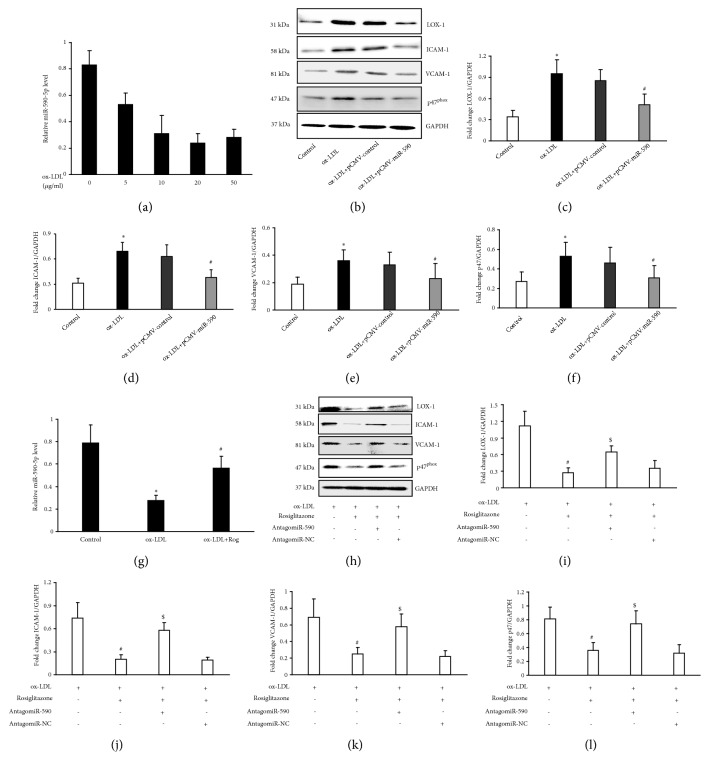
*miR-590-5p is required for the suppression of LOX-1 and LOX-1-dependent inflammatory and oxidative injury in HUVECs induced by ox-LDL*. (a) The expressions of miR-590-5p were determined in HUVECs stimulated by ox-LDL at a range of doses (0~50 *μ*g/mL). (b–f) The pMIR-CMV-miR590 and control plasmid were, respectively, transfected into HUVECs 48 h before the cells were stimulated by 20 *μ*g/mL ox-LDL; the protein levels of LOX-1, ICAM-1, VCAM-1, and p47^phox^ were detected and quantified by Western blot analysis. (g) The expression of miR-590-5p in HUVECs stimulated by ox-LDL with or without Rosiglitazone (50 *μ*mol/mL). (h–l) The effect of antagomiR-590-5p and the negative control (antagomiR-NC) on Rosiglitazone-induced protein levels of LOX-1, ICAM-1, VCAM-1, and p47^phox^ in HUVECs stimulated by ox-LDL (n=4, *∗* P<0.05; vs Control group; n=4, # P<0.05 vs. ox-LDL-induced group; $ P<0.05 vs. ox-LDL together with Rog treated group).

Previous studies indicated that LOX-1 could be negatively regulated by miR-590-5p; therefore we determined the expression of miR-590-5p in ox-LDL-induced human umbilical vein endothelial cells (HUVECs). As shown in Figure 2, the baseline level of miR-590-5p was high in* in vitro* cultured HUVECs, but it gradually decreased in HUVECs after stimulating by dose-dependent ox-LDL (Figure 2(a)). To confirm the role of miR-590-5p in regulating LOX-1, we transfected the miR-590-5p expression plasmid (pMIR-CMV-miR590, Vigene Bioscience) into HUVECs and then examined the cellular LOX-1 after stimulating the cells with ox-LDL (20 *μ*g/ml) for 24 h. Compared with the effect of control plasmid, miR-590-5p overexpression prevented the upregulation of LOX-1 in ox-LDL-induced HUVECs (Figures 2(b) and 2(c)). In addition, ICAM-1, VCAM-1, and p47^phox^ were all suppressed in ox-LDL-induced HUVECs transfecting by miR-590-5p expression plasmid (Figures 2(b), 2(d), 2(e), and 2(f)). Importantly, the level of miR-590-5p in HUVECs repressed by ox-LDL was rebounded after pretreating the cells with Rosiglitazone (Figure 2(g)). To determine whether PPAR-*γ* activation inhibited LOX-1 through regulating miR-590-5p, we suppressed the expression of miR-590-5p in HUVECs by introducing antagomiR-590-5p. Compared with the effect of antagomiR-NC, antagomiR-590-5p pretreatment maintained ox-LDL-induced LOX-1 expression in Rosiglitazone-pretreated HUVECs (Figures 2(h) and 2(i)), and Rosiglitazone also failed to suppress ox-LDL-induced ICAM-1, VCAM-1, and p47^phox^ in HUVECs after blocking miR-590-5p (Figures 2(h), 2(j), 2(k), and 2(l)), indicating that miR-590-5p is required for Rosiglitazone-mediated suppression of LOX-1 and LOX-1 mediated endothelial injury.

### 3.3. Rosiglitazone Upregulated miR-590-5p in HUVECs by Activating PPAR-*γ* and Promoting Its Nuclear Translocation

**Figure 3:**
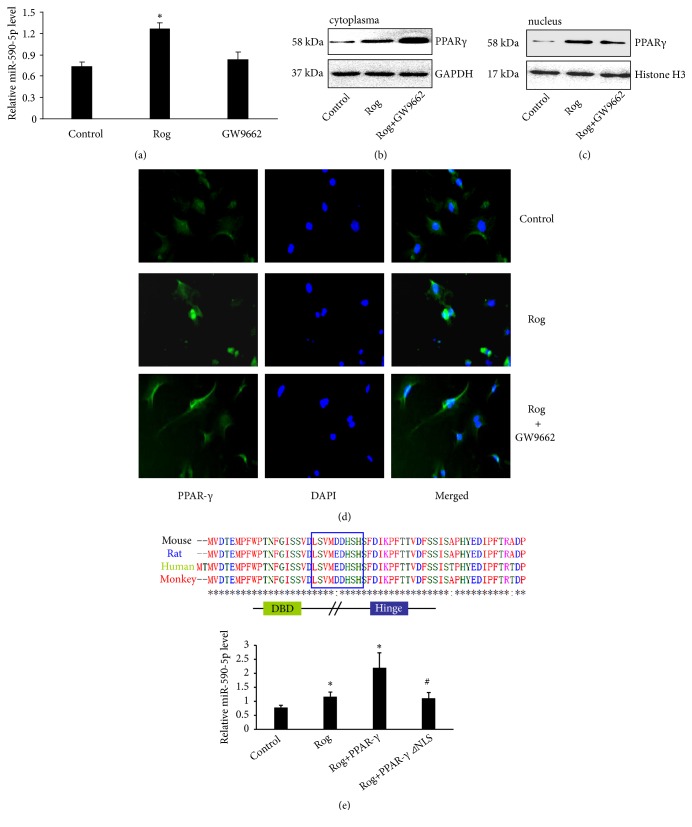
*miR-590-5p transcription is regulated by nuclear translocated PPAR-γ*. Preferential induction the HUVECs with or without GW9662 (5 *μ*mol/L) for 1 h followed by the induction with Rosiglitazone (50 *μ*mol/L) for 24 h and then determined (a) the expression of miR-590-5p, (b) the cytoplasm PPAR-*γ* protein level, and (c) the nucleus PPAR-*γ* protein level and (d) immunofluorescent staining of HUVECs using anti-PPAR-*γ* antibody (green) and dapi staining (purple DNA dye). (e) The expressions of miR-590-5p were determined in HUVECs induced by Rosiglitazone and Rosiglitazone-induced HUVECs transfected with PPAR-*γ* or PPAR-*γ*^*⊿*NLS^ plasmid lacking the nuclear localization signal (NLS) (n=3, *∗* P<0.05; vs. Control group; # P<0.05 vs. PPAR-*γ* with Rog treated group).

Next, we investigated whether PPAR-*γ* activation is essential to Rosiglitazone-mediated upregulation of miR-590-5p in endothelial cells. In vitro cultured HUVECs were pretreated with or without PPAR-*γ* antagonist GW9662 (5 *μ*mol/L) for 1 h and followed by Rosiglitazone treatment for 24 h, then the expression of miR-590-5p was detected in cells. The results showed that miR-590-5p level significantly increased in HUVECs with Rosiglitazone, but it did not increase after being pretreated with GW9662 (Figure 3(a)). In addition, we found that both cytoplasm and nuclear PPAR-*γ* were markedly enhanced in HUVECs after stimulating with Rosiglitazone, but the nuclear translocation of PPAR-*γ* attenuated in GW9662 pretreated HUVECs (Figures 3(b) and 3(c)). The effect of GW9662 on blocking PPAR-*γ* nuclear translocation was also confirmed by immunostaining (Figure 3(d)). To further confirm the function of PPAR-*γ* nuclear translocation in regulating miR-590-5p, we transfected the WT or the mutant type of PPAR-*γ* plasmid (PPAR-*γ*^*⊿*NLS^), which lacked the nuclear localization signal (NLS), respectively, into HUVECs (Figure 3(e)). In HUVECs transfected by WT-PPAR-*γ*, miR-590-5p expression increased by 2-fold (n=3, P < 0.01), compared with that transfected by PPAR-*γ*^*⊿*NLS^ after stimulating with Rosiglitazone (Figure 3(e)), suggesting that the nuclear localization of PPAR-*γ* is critically involved in Rosiglitazone-mediated activation of miR-590-5p.

### 3.4. Nuclear PPAR-*γ* Activates the Transcription of miR-590-5p through Binding to Its Promoter

**Figure 4:**
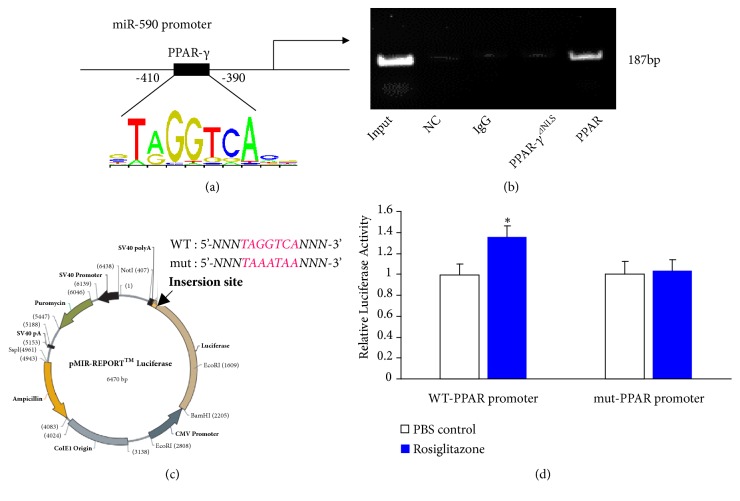
*Nuclear PPAR-γ activates the transcription of miR-590-5p through binding to its promoter*. (a) A schematic diagram depicting the binding sites for PPAR-*γ* in the promoter of miR-590-5p. (b) ChIP assays were performed with PPAR-*γ* antibody and the miR-590-5p promoter region with the PPAR-*γ* binding motif was amplified by PCR. (c) A schematic representation of the insertion of WT-PPAR-*γ* or mut-PPAR-*γ* DNA binding motif, locating at miR-590-5p promoter region into the pMIR-REPORT Luciferase plasmid. (d) HEK-293T cells were transfected with a renilla luciferase plasmid plus a luciferase plasmid containing WT-PPAR-*γ* or mut-PPAR-*γ* promoter region; then the cells were stimulated by Rosiglitazone (PBS as negative control) for 24 h. Luciferase activities were determined by the ratio of pMIR-REPORT plasmid to pCMV-Renilla control (n=3, *∗* P<0.05).

The involvement of PPAR-*γ* in regulating miR-590-5p expression prompted us to think whether miR-590-5p is one of the transcriptional targets of PPAR-*γ*. Therefore, we screened the location of miR-590-5p in miRBase database (http:mirbase.org/). In the promoter region (-2000bp~-100bp) of miR-590-5p, we found one predictable sequence-specific binding site for PPAR-*γ* (*TAGGTCA*) locating at -410~-390 bp of the upstream of miR-590-5p by JASPAR computational program database (Figure 4(a)). To test this hypothesis, we performed chromatin immunoprecipitation (ChIP)-PCR assay to examine protein-DNA interactions at the miRNA promoters in HUVECs transfer with WT-PPAR or PPAR-*γ*^*⊿*NLS^ plasmids. The promoter region (-410~-390 bp) of miR-590-5p was determined by PCR in the anti-PPAR-*γ* immunoprecipitated DNA. The recruitment of PPAR-*γ* to this region was only observed in PPAR-*γ* transfected but not in PPAR-*γ*^*⊿*NLS^ transfected cells (Figure 4(b)). Next, the PPAR-*γ* binding motif was mutated and cloned into the pMIR-REPORT Luciferase plasmid, both WT- and mut-type of PPAR-*γ* promoters were transfected into HEK-293T cells, respectively (Figure 4(c)), and cells were stimulated by Rosiglitazone at 48 h after transfection. The result of luciferase expression activity suggested that gene transcription could be driven by the nuclear recruitment of PPAR-*γ* to this region of the miR-590-5p promoter (Figure 4(d)).

## 4. Discussion

This study demonstrates that Rosiglitazone treatment effectively protects the high-fat diet-induced ApoE^−/−^ mice through reducing the occurrence and development of atherosclerosis. During the process of atherosclerosis, LOX-1 upregulation plays a crucial role in the uptake of ox-LDL in endothelium of the arterial wall. Here, we reveal that Rosiglitazone-mediated PPAR-*γ* activation suppresses the expression of LOX-1 in ox-LDL stimulated HUVECs via activating the transcription of miR-590-5p.

Rosiglitazone belongs to the family of Thiazolidinedione (TZD), which is widely used in the treatment of type 2 diabetes, inflammation, and atherosclerosis. Emerging evidence indicated that LOX-1 could be negatively regulated by PPAR-*γ* activation in the treatment of atherosclerosis with TZDs [9, 22]. LOX-1 mediated suppression of ABCA1 in macrophages could be reversed by TZDs, which improved the reverse cholesterol transport and prevented the foam cell formation and ox-LDL accumulation on the arterial wall in the development of atherosclerosis [23, 24]. However, the precise role of PPAR-*γ* in ox-LDL-induced endothelial cells remained unclear. We previously revealed that Ciglitazone treatment also suppressed LOX-1 in ox-LDL-induced endothelial cells and improved angiogenesis by activating PPAR-*γ* [25]. In this study, we further confirmed that PPAR-*γ* activation could inhibit the upregulation of LOX-1 and LOX-1 mediated oxidative stress and adhesion of inflammatory cells by targeting miR-590-5p.

In the present study, we found that miR-590-5p was significantly downregulated in ox-LDL-stimulated HUVECs in a dose-dependent manner. Overexpression of miR-590-5p significantly reversed LOX-1 expression and repressed LOX-1 mediated protein levels of ICAM-1, VCAM-1, and p47^phox^ in ox-LDL-induced HUVECs. The inhibitory role of miR-590-5p on LOX-1 signaling was observed in both ox-LDL-induced and AngII-induced HUVECs [11, 26]. miR-590-5p mimics attenuated cell apoptosis, reduced ROS production, and improved angiogenesis through targeting the 3'-UTR of LOX-1 gene [11]. Consistent with these results, we further revealed the upstream regulator of miR-590-5p. The activity of microRNAs could be extensively regulated by the nuclear transcription factors. PPAR-*γ*, also named NR1C3 (nuclear receptor subfamily 1, group C, member 3), is regarded as one kind of transcription factors encoded by the PPARG gene. In the proximal promoter region of miR-590-5p, we observed one genetically conserved binding site for PPAR-*γ* [27, 28]. This conserved core sequence failed to be detected by ChIP-PCR assay in PPAR-*γ*^*⊿*NLS^ transfected HUVECs after stimulating with Rosiglitazone, suggesting that PPAR-*γ* nuclear translocation is required for its binding with the DNA promoter of miR-590-5p. In the previous studies, Rosiglitazone treatment increased the nuclear expression of PPAR-*γ* in bone marrow stem cell (BMSC) and ECs, which promoted cell differentiation and adipogenesis [29, 30]. We also reported the event of PPAR-*γ* nuclear translocation in Ciglitazone-pretreated dendritic cells [31]. Nuclear translocated PPAR-*γ* can be either an activator or a repressor of gene transcription. For example, PPAR-*γ* is required to mediate the antioxidant effect and promotes the production of antioxidant properties in the endothelium by forming a transcriptional complex with retinol binding protein 7 (RBP7) [32]. PPAR-*γ* activation by Rosiglitazone also blocks NFATc1 from binding to its own promoter [33]. In this study, we did not test the possibility whether nuclear translocation of PPAR-*γ* promoted the transcription of miR-590-5p in coordination with the other transcriptional factors. But Rosiglitazone induced miR-590-5p promoter activity significantly attenuated in HUVECs after mutating the DNA binding site for PPAR-*γ*, indicating a function role of nuclear PPAR-*γ* in regulating miR-590-5p transcription.

Previous studies showed a critical role of PPAR-*γ* in preventing insulin resistance and dysregulation of lipid metabolism through regulating microRNA transcription [34–36]. Multiple microRNAs, such as miR-125a, miR-424, and miR-503, that are transcriptionally activated by PPAR-*γ* are also found in inflammation-mediated angiogenesis in endothelial cells [15, 16]. Here, we observed that miR-590-5p could be regulated by PPAR-*γ* in HUVECs in a Rosiglitazone-dependent manner, which effectively suppressed LOX-1 and LOX-1 mediated inflammation and oxidative stress. Our findings raise the possibility that one kind of TZD drugs commonly used in diabetic therapy also has tremendous therapeutic potential in treatment of endothelial injury related atherosclerosis.

## Data Availability

The data used to support the findings of this study are available from the corresponding author upon request.
